# Non-linear connection between the triglyceride–glucose index and prediabetes risk among Chinese adults: a secondary retrospective cohort study

**DOI:** 10.1186/s40001-024-02121-x

**Published:** 2024-11-05

**Authors:** Changchun Cao, Yong Han, Huanhua Deng, Xiaohua Zhang, Haofei Hu, Fubing Zha, Yulong Wang

**Affiliations:** 1 Department of Rehabilitation, Shenzhen Second People’s Hospital Dapeng New District Nan’ao Hospital, No. 6, Renmin Road, Dapeng New District, Shenzhen, 518000 Guangdong China; 2 Department of Emergency, Shenzhen Second People’s Hospital, The First Affiliated Hospital of Shenzhen University, Shenzhen, 518000 Guangdong China; 3 Department of Nephrology, Shenzhen Second People’s Hospital, The First Affiliated Hospital of Shenzhen University, No.3002, Sungang West Road, Futian District, Shenzhen, 518000 Guangdong China; 4 Department of Rehabilitation, Shenzhen Second People’s Hospital, The First Affiliated Hospital of Shenzhen University, No.3002, Sungang West Road, Futian District, Shenzhen, 518000 Guangdong China

**Keywords:** Triglyceride–glucose index, Triglyceride, Fasting plasma glucose, Prediabetes, Non-linearity

## Abstract

**Background:**

The triglyceride–glucose (TyG) index has garnered recognition as a surrogate marker for insulin resistance, a pivotal factor in the pathogenesis of various metabolic disorders. Despite its emerging role, the empirical evidence delineating its association with prediabetes mellitus (Pre-DM) remains scant. This research aims to clarify the link between the TyG index and the likelihood of Pre-DM development within a Chinese demographic.

**Methods:**

This investigation was structured as a retrospective cohort analysis, encompassing a sample of 179,177 Chinese adults. These individuals underwent medical examinations at the Rich Healthcare Group over a period spanning from 2010 to 2016. To ascertain the relationship between the TyG index and the incidence of Pre-DM, this study employed Cox regression analysis complemented by sensitivity and subgroup assessments. Furthermore, Cox proportional hazards regression with cubic spline functions and smooth curve fitting was incorporated to explore the existence of any non-linear connection within this association.

**Results:**

Upon adjusting for a comprehensive array of confounding variables, a statistically significant positive correlation between the TyG index and the risk of Pre-DM was identified (HR: 1.60, 95%CI 1.56–1.65, *P* < 0.001). The analysis illuminated a non-linear relationship, with an inflection point at a TyG index value of 8.78. For TyG index values below and above this inflection point, the HR was calculated to be 1.94 (95%CI 1.86–2.03) and 1.26 (95%CI 1.20–1.33), respectively. Sensitivity analyses further fortified the reliability of these findings.

**Conclusions:**

This comprehensive examination delineated a significantly positive, non-linear correlation between the TyG index and the risk of Pre-DM within a Chinese population. Individuals with TyG index values below 8.78 have a significantly increased risk of developing prediabetes. These findings underscore the TyG index’s potential efficacy as a predictive tool for assessing Pre-DM risk in clinical practice.

**Supplementary Information:**

The online version contains supplementary material available at 10.1186/s40001-024-02121-x.

## Introduction

Prediabetes (Pre-DM), characterized by elevated blood glucose levels that do not meet the criteria for diabetes, is a significant public health issue [1]. In China, the prevalence of prediabetes was reported at 35.7% in 2013, with an estimated 5–10% of these individuals progressing to diabetes annually [2, 3]. The condition is associated with an increased risk of macrovascular and microvascular complications, underscoring the importance of early identification and intervention [4–6].

Insulin resistance (IR) plays a critical role in the pathogenesis of various metabolic disorders, including diabetes mellitus, non-alcoholic fatty liver disease (NAFLD), obesity, metabolic syndrome, and prediabetes [7–10]. While accurate, the hyperinsulinemic–euglycemic clamp technique is limited by its complexity and cost [11]. As an alternative, the triglyceride–glucose (TyG) index, based on fasting plasma glucose and triglyceride levels, offers a simpler and cost-effective method for assessing IR [12, 13]. Initial evidence of the utility of the TyG index was provided by a foundational prospective cohort study, which, despite its relatively modest cohort size of 4543 participants, demonstrated a significant positive correlation between the TyG index and the subsequent onset of Pre-DM [14]. This preliminary finding was further substantiated by a subsequent prospective cohort study, which expanded the sample size to 7953 subjects and reinforced the robust association between the TyG index and the increased risk of Pre-DM [15]. Notwithstanding the promising outcomes of these preliminary investigations into the relationship between the TyG index and Pre-DM, it is imperative to acknowledge that the scale of these studies was relatively limited. Addressing this gap in the literature, this study, encompassing a substantial cohort of 179,177 Chinese participants, endeavors to elucidate more definitively the relationship between the TyG index and the risk of developing Pre-DM.

## Methods

### Data source

The data set utilized in this study was sourced from the DATADRYAD platform, a digital repository that allows researchers to access and download a wealth of raw data freely. This study utilized the DATADRYAD platform to access the data set originally uploaded by Chen et al. [16], which contains data on 211,833 Chinese individuals. In compliance with Dryad’s terms of service, this study performed a secondary analysis on this publicly available data set.

### Study population

The initial research received approval from the Rich Healthcare Group Review Board, so no additional ethical approval was necessary for this secondary analysis. The initial investigation and this study were conducted per the principles set forth in the Declaration of Helsinki, and all protocols complied with applicable guidelines and regulations.

**Fig. 1:**
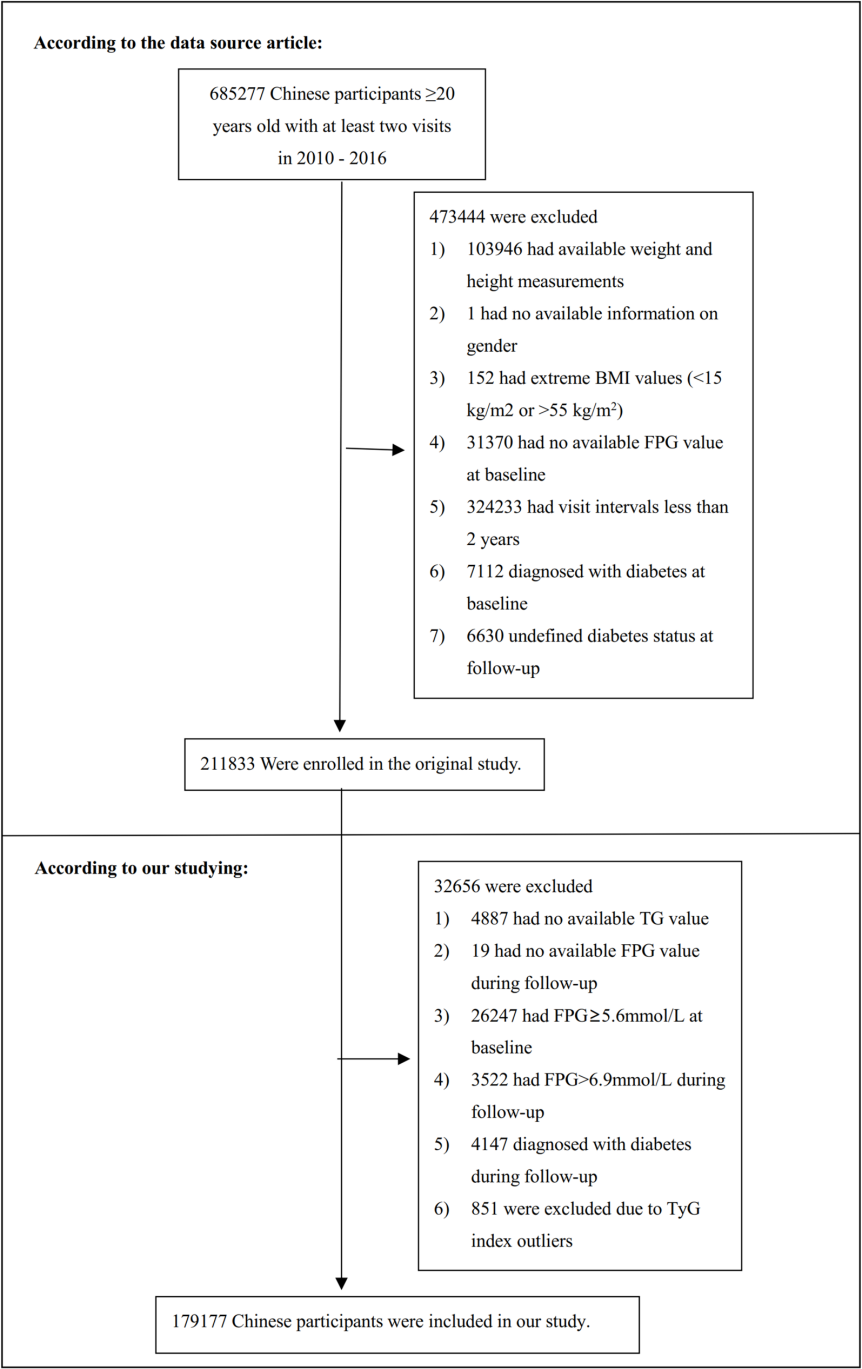
Study population

The initial investigation enrolled 685,277 Chinese individuals who were older than 20 years and had undergone at least two medical assessments. This encompassed 32 locations and 11 urban areas within China. The exclusion criteria consisted of the following: (1) having been diagnosed with diabetes at the beginning of the study and during subsequent check-ups; (2) an undefined diabetes status during follow-up; (3) extreme (BMI) values (< 15 kg/m^2^ or > 55 kg/m^2^); (4) incomplete data regarding weight, height, gender, triglyceride (TG), or fasting plasma glucose (FPG) at the start of the study, or FPG during follow-up; (5) having an FPG level surpassing 5.6 mmol/L at the beginning and exceeding 6.9 mmol/L during follow-up; and (6) having a follow-up duration of less than two years. Ultimately, the study encompassed 179,177 participants. The research’s design and procedures are delineated in Fig. 1.

### Data collection

Data collection was conducted in a standardized setting by well-trained staff to ensure consistency across all measurements. The team gathered demographic data, including age, systolic and diastolic blood pressure (SBP and DBP), height, and weight. Height and weight measurements were taken with participants in light clothing and without shoes, and BMI was calculated using the formula kg/m^2^. Blood pressure readings were obtained using a standard mercury sphygmomanometer. Furthermore, clinical parameters such as low-density lipoprotein cholesterol (LDL-C), alanine aminotransferase (ALT), FPG, TG, serum creatinine (Scr), total cholesterol (TC), aspartate aminotransferase (AST), blood urea nitrogen (BUN), and high-density lipoprotein cholesterol (HDL-C) were measured using a Beckman 5800 autoanalyzer.

### TyG index

The TyG index was determined by applying the formula: Ln[FPG (mg/dL)) × (TG (mg/dL)/2) [13].

### Definition

Pre-DM was delineated as the presence of impaired FPG levels, specifically within the range of 5.6–6.9 mmol/L [17].

### Statistical analysis

Statistical analyses within this study were meticulously performed utilizing R software in conjunction with Empower Stats. This study stratified the TyG index into quartiles for analysis. For normally distributed continuous variables, this study reported means and standard deviations, whereas medians and interquartile ranges were presented for those with skewed distributions. Categorical variables were summarized using percentages. This study compared continuous variables with either one-way ANOVA or the Kruskal–Wallis. Categorical variables were assessed using the chi-square test. Survival and cumulative event rates were evaluated using the Kaplan–Meier method, with differences among groups tested by the log-rank test. In addition, this study calculated hazard ratios (HR) for adverse events using Kaplan–Meier estimates.

Owing to the substantial proportion of missing data for AST, smoking status, and drinking status, this study initially categorized the AST variables into tertiles. Subsequently, the missing values for smoking status, drinking status, and AST were classified into a distinct category designated as the ‘Not recorded group’. This study had some missing data, including HDL-C, SBP, TC, BUN, ALT, DBP, Scr, and LDL-C. The prevalence of missing data for each parameter is enumerated as follows: SBP and DBP both exhibited omissions in 0.009% of cases (16 and 17 individuals, respectively), TC in 0.001% (1 individual), HDL-C in 43.935% (78,704 individuals), LDL-C in 43.583% (78,113 individuals), ALT in 0.787% (1,410 individuals), BUN in 8.931% (16,005 individuals), and Scr in 4.572% (8193 individuals). This investigation employed the technique of multiple imputations to address the issue of missing data [18], thereby mitigating the potential variability introduced by absent variables. The imputation model adopted for this purpose was characterized by a linear regression framework executed over ten iterations. The variables incorporated into the model encompassed a comprehensive set of demographic and clinical parameters: sex, family history of diabetes, age, HDL-C, SBP, drinking status, TC, BUN, ALT, DBP, Scr, smoking status, AST, and LDL-C. The analytical approach to handling missing data was predicated on the Missing-at-Random (MAR) assumptions [19], a methodology that assumes the missingness of data is related to the observed data but not the missing data itself.

This study explored the relationship between the TyG index and the likelihood of developing Pre-DM by employing both univariate and multivariate Cox proportional-hazards regression analyses following a collinearity assessment. The analysis framework comprised three distinct models: Model 1, which was unadjusted; Model 2, which was controlled for family history of diabetes, BMI, age, drinking status, DBP, sex, smoking status, and SBP; and Model 3, which was controlled for HDL-C, BUN, ALT, Scr, LDL-C, and AST, alongside the variables adjusted in Model 2. Throughout the study, this study meticulously documented HR and 95% confidence intervals (CI). In addition, the collinearity assessment excluded TC from the final multivariate Cox proportional hazards regression equation due to its collinearity with other assessed factors, as detailed in Supplementary Table S1.

Notably, higher rates of Pre-DM were observed among older adults and individuals with obesity. To delve deeper into the association between the TyG index and the risk of prediabetes, this study conducted sensitivity analyses, excluding participants aged 65 years or older or those with a BMI of 25 kg/m^2^ or higher. A generalized additive model (GAM) was employed to validate the findings, allowing for the inclusion of continuous variables as curves within the model. Furthermore, this study computed *E* values to investigate the potential for unmeasured confounding factors that might influence the observed link between the TyG index and prediabetes risk [20].

To investigate the potential non-linear association between the TyG index and the risk of Pre-DM, the analysis employed Cox proportional hazards regression, incorporating cubic spline functions and smooth curve fitting. In instances where non-linearity was detected, the inflection point was determined by recursive algorithms. Subsequently, this study applied a two-piecewise Cox proportional hazards regression approach to ascertain the threshold effect of the TyG index on Pre-DM incidence, guided by the insights from the smoothed curve analysis.

To further dissect the data, this study applied the Cox proportional hazard model to various subgroups, including family history of diabetes, DBP, age, smoking status, BMI, SBP, drinking status, and sex. This study categorized these subgroups based on clinically relevant thresholds: DBP (< 90, ≥ 90 mmHg), BMI (< 25, ≥ 25 kg/m^2^), age (< 65, ≥ 65 years), and SBP (< 140, ≥ 140 mmHg). Each stratification underwent a comprehensive analysis with full adjustments. To assess the interactions among subgroups, this study utilized the likelihood ratio test. The documentation and presentation of all findings within this study were meticulously aligned with the guidelines delineated in the STROBE statement [21]. Values of *P* ≤ 0.05 were deemed to indicate statistical significance.

## Results

### Baseline characteristics of participants

This study included 179,177 participants who were free from Pre-DM at baseline. The mean age was 41.12 ± 12.14 years, with males comprising 53.09%. Over an average follow-up period of 3.14 years, 20,248 participants developed Pre-DM. Key demographics, laboratory tests, and other relevant data are summarized in Table 1. Participants were categorized into four quartiles based on their TyG index values (Q1 ≤ 7.89; 7.89 < Q2 ≤ 8.27; 8.27 < Q3 ≤ 8.69; Q4 > 8.69). Analysis revealed that the highest quartile (Q4) exhibited elevated levels of age, SBP, DBP, BMI, AST, ALT, TG, LDL-C, TC, BUN, Scr, and FPG compared to the other quartiles. This group also had a higher prevalence of males, current smokers and drinkers, and a family history of diabetes. Conversely, the first quartile (Q1) was characterized by higher levels of HDL-C in comparison to its counterparts.

**Table 1:** Baseline characteristics of participants

TyG index	Q1(≤ 7.89)	Q2(7.89 to ≤ 8.27)	Q3(8.27 to ≤ 8.69)	Q4(> 8.69)	***P*** value
Participants	44,782	44,802	44,796	44,797	
Gender					
Male	14,177 (31.66%)	20,691 (46.18%)	26,808 (59.84%)	33,450 (74.67%)	< 0.001
Female	30,605 (68.34%)	24,111 (53.82%)	17,988 (40.16%)	11,347 (25.33%)	
Age(years)	37.04 ± 9.66	39.79 ± 11.55	42.58 ± 12.81	45.08 ± 12.77	< 0.001
Smoking status					
Current-smoker	884 (1.97%)	1714 (3.83%)	2671 (5.96%)	4042 (9.02%)	< 0.001
Ex-smoker	261 (0.58%)	444 (0.99%)	628 (1.40%)	724 (1.62%)	
Never-smoker	9435 (21.07%)	9871 (22.03%)	9748 (21.76%)	9037 (20.17%)	
Not recorded					
Drinking status					
Current-drinker	90 (0.20%)	175 (0.39%)	261 (0.58%)	447 (1.00%)	< 0.001
Ex-drinker	1036 (2.31%)	1515 (3.38%)	2023 (4.52%)	2644 (5.90%)	
Never-drinker	9454 (21.11%)	10,339 (23.08%)	10,763 (24.03%)	10,712 (23.91%)	
Not recorded	34,202 (76.37%)	32,773 (73.15%)	31,749 (70.87%)	30,994 (69.19%)	
Family history of diabetes					
No	43,974 (98.20%)	43,897 (97.98%)	43,892 (97.98%)	43,877 (97.95%)	0.028
Yes	808 (1.80%)	905 (2.02%)	904 (2.02%)	920 (2.05%)	
SBP (mmHg)	112.01 ± 13.83	115.65 ± 14.93	119.53 ± 15.71	124.20 ± 16.11	< 0.001
DBP (mmHg)	69.81 ± 9.48	72.00 ± 9.94	74.46 ± 10.40	77.85 ± 10.83	< 0.001
BMI (kg/m2)	21.20 ± 2.57	22.23 ± 2.90	23.45 ± 3.10	25.12 ± 3.07	< 0.001
ALT (U/L)	16.78 ± 16.34	19.87 ± 20.05	24.00 ± 21.83	32.29 ± 24.57	< 0.001
AST					
Low	8595 (19.19%)	7336 (16.37%)	5650 (12.61%)	3427 (7.65%)	< 0.001
Medium	6461 (14.43%)	6333 (14.14%)	6407 (14.30%)	5650 (12.61%)	
High	3828 (8.55%)	4934 (11.01%)	6605 (14.74%)	9651 (21.54%)	
Not recorded	25,898 (57.83%)	26,199 (58.48%)	26,134 (58.34%)	26,069 (58.19%)	
HDL-C (mmol/L)	1.45 ± 0.30	1.41 ± 0.30	1.36 ± 0.29	1.28 ± 0.29	< 0.001
TG (mmol/L)	0.57 ± 0.13	0.87 ± 0.13	1.25 ± 0.19	2.41 ± 1.21	< 0.001
LDL-C (mmol/L)	2.41 ± 0.57	2.60 ± 0.61	2.79 ± 0.66	2.97 ± 0.71	< 0.001
TC (mmol/L)	4.27 ± 0.74	4.52 ± 0.79	4.77 ± 0.85	5.12 ± 0.92	< 0.001
BUN (mmol/L)	4.52 ± 1.16	4.55 ± 1.18	4.64 ± 1.17	4.74 ± 1.15	< 0.001
Scr (umol/L)	64.35 ± 13.92	67.96 ± 16.21	71.22 ± 15.77	74.86 ± 15.05	< 0.001
FPG (mmol/L)	4.58 ± 0.50	4.74 ± 0.46	4.83 ± 0.44	4.94 ± 0.42	< 0.001
TyG index	7.61 ± 0.22	8.08 ± 0.11	8.47 ± 0.12	9.08 ± 0.35	< 0.001

Values are n (%) or mean ± standard deviation or medians (quartile interval)

*TyG index* triglyceride–glucose index, *SBP* systolic blood pressure, *DBP* diastolic blood pressure, *BMI* body mass index, *ALT* alanine aminotransferase, *AST* aspartate aminotransferase, *HDL-C* high-density lipoprotein cholesterol, *LDL-C* low-density lipoprotein cholesterol, *TC* total cholesterol, *TG* triglycerides, *Scr* serum creatinine, *BUN* blood urea nitrogen, *FPG* fasting plasma glucose

### The incidence rate of Pre-DM

Table 2 outlines the prevalence of Pre-DM among 179,177 participants throughout the study period. Overall, the incidence rate was 11.30% (range: 11.15–11.45%). Specifically, the incidence rates across the four TyG index quartiles were as follows: 5.99% (5.77–6.21%) for the first quartile, 8.55% (8.29–8.81%) for the second, 12.14% (11.83–12.44%) for the third, and 18.53% (18.17–18.89%) for the fourth. Moreover, the cumulative incidence rates per 100,000 person-years for the overall population and the respective TyG index quartiles were 3598.90, 1842.77, 2722.52, 3915.24, and 5995.40, respectively. Individuals in higher TyG index quartiles had higher incidence and cumulative incidence rates of Pre-DM than those in lower quartiles.

**Table 2:** Incidence rate of prediabetes

TyG index	Participants (n)	Prediabetes events (n)	Cumulative incidence (95%CI) (%)	Per 100,000 person-year
Total	179,177	20,248	11.30 (11.15–11.45)	3598.90
Q1	44,782	2682	5.99 (5.77–6.21)	1842.77
Q2	44,802	3830	8.55 (8.29–8.81)	2722.52
Q3	44,796	5437	12.14 (11.83–12.44)	3915.24
Q4	44,797	8299	18.53 (18.17–18.89)	5995.40
*P* for trend			< 0.001	< 0.001

**Fig. 2:**
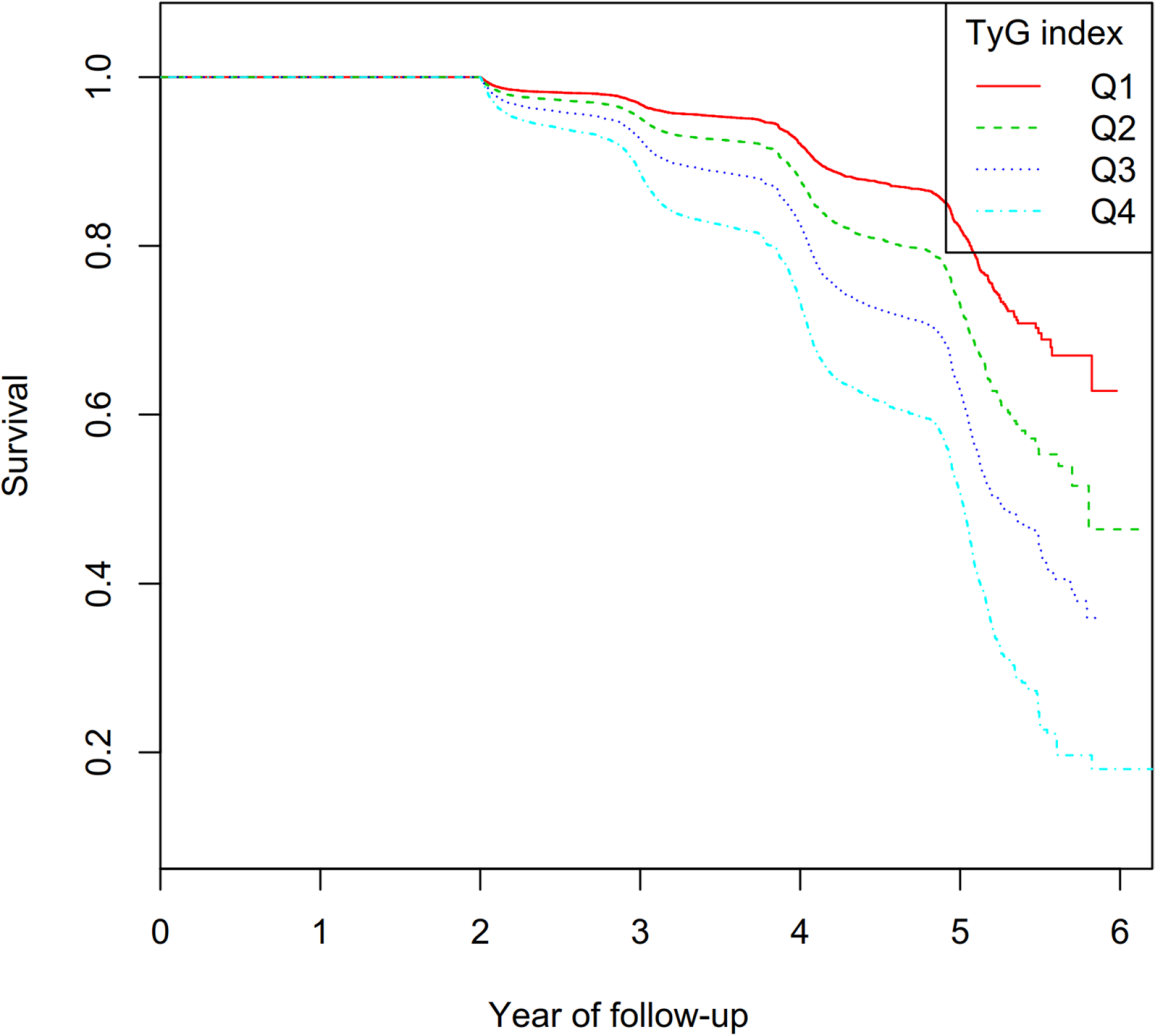
Kaplan–Meier event-free survival curve. Kaplan–Meier analysis of incident prediabetes based on TyG index quartiles (log-rank, *P* < 0.0001)

Figure 2 presents Kaplan–Meier curves illustrating the likelihood of surviving without Pre-DM. There was a significant difference in the risk of developing Pre-DM among the four TyG index groups (*P* < 0.001), with a clear trend showing that as TyG index values rose, the likelihood of surviving without Pre-DM gradually decreased. This suggested that participants with the highest TyG index had the highest risk of Pre-DM onset.

### Univariate analysis

Table 3 displays the outcomes of the univariate analysis. It revealed a positive correlation between the risk of developing Pre-DM and several factors: age, SBP, DBP, BMI, AST, ALT, TG, LDL-C, TC, BUN, Scr, and FPG. Conversely, HDL-C showed a negative relationship with Pre-DM risk. In addition, individuals who never drink or never smoke were found to have a lower risk of Pre-DM. The analysis indicated that females have a lower risk of Pre-DM than men.

**Table 3:** Results of the univariate analysis

	Statistics	HR (95%CI)	*P* value
Gender			< 0.001
Male	95,126 (53.09%)	ref	
Female	84,051 (46.91%)	0.64 (0.62, 0.66)	
Age(years)	41.12 ± 12.14	1.03 (1.03, 1.03)	< 0.001
Smoking status			
Current-smoker	9311 (5.20%)	ref	
Ex-smoker	2057 (1.15%)	0.80 (0.70, 0.91)	0.001
Never-smoker	38,091 (21.26%)	0.72 (0.68, 0.76)	< 0.001
Not recorded	129,718 (72.40%)	0.77 (0.73, 0.82)	< 0.001
Drinking status			
Current-drinker	973 (0.54%)	ref	
Ex-drinker	7218 (4.03%)	0.63 (0.54, 0.75)	< 0.001
Never-drinker	41,268 (23.03%)	0.58 (0.49, 0.68)	< 0.001
Not recorded	129,718 (72.40%)	0.59 (0.51, 0.69)	< 0.001
Family history of diabetes			0.437
No	175,640 (98.03%)	ref	
Yes	3537 (1.97%)	1.04 (0.95, 1.13)	
SBP (mmHg)	117.85 ± 15.83	1.03 (1.02, 1.03)	< 0.001
DBP (mmHg)	73.53 ± 10.61	1.03 (1.03, 1.03)	< 0.001
BMI (kg/m2)	23.00 ± 3.26	1.12 (1.12, 1.13)	< 0.001
ALT (U/L)	23.24 ± 21.71	1.00 (1.00, 1.00)	< 0.001
AST			
Low	25,008 (13.96%)	ref	
Medium	24,851 (13.87%)	1.14 (1.08, 1.20)	< 0.001
High	25,018 (13.96%)	1.51 (1.44, 1.59)	< 0.001
Not recorded	104,300 (58.21%)	0.84 (0.81, 0.88)	< 0.001
HDL-C (mmol/L)	1.38 ± 0.30	0.79 (0.75, 0.83)	< 0.001
TG (mmol/L)	1.28 ± 0.93	1.20 (1.19, 1.21)	< 0.001
LDL-C (mmol/L)	2.69 ± 0.67	1.28 (1.25, 1.30)	< 0.001
TC (mmol/L)	4.67 ± 0.88	1.22 (1.20, 1.24)	< 0.001
BUN (mmol/L)	4.61 ± 1.17	1.14 (1.12, 1.15)	< 0.001
Scr (umol/L)	69.59 ± 15.75	1.01 (1.01, 1.01)	< 0.001
FPG (mmol/L)	4.77 ± 0.48	5.79 (5.58, 6.00)	< 0.001
TyG index	8.31 ± 0.58	2.12 (2.08, 2.17)	< 0.001

*TyG index* triglyceride–glucose index, *SBP* systolic blood pressure, *DBP* diastolic blood pressure, *BMI* body mass index, *ALT* alanine aminotransferase, *AST* aspartate aminotransferase, *HDL-C* high-density lipoprotein cholesterol, *LDL-C* low-density lipoprotein cholesterol, *TC* total cholesterol, *TG* triglycerides, *Scr* serum creatinine, *BUN* blood urea nitrogen, *FPG* fasting plasma glucose

### The relationship between TyG index and Pre-DM

As the TyG index met the proportional hazards assumption, the association between TyG index and prediabetes risk was evaluated by the Cox proportional hazards regression model. Table 4 outlines the results from Cox proportional hazard regression models, detailing HR and 95% CI for the relationship between the TyG index and the risk of developing Pre-DM. In Model 1, the HR for the TyG index's correlation with Pre-DM was 2.12 (95%CI 2.08–2.17). Model 2, which was controlled for family history of diabetes, BMI, age, drinking status, DBP, sex, smoking status, and SBP, showed an HR of 1.56 (95%CI 1.52–1.60). Model 3, which was further adjusted for HDL-C, BUN, ALT, Scr, LDL-C, and AST, presented an HR of 1.60 (95%CI 1.56–1.65). These findings indicated a 60% increase in Pre-DM risk for each unit increment in the TyG index.

**Table 4:** Relationship between TyG index and incident prediabetes in different models

Variable	Model 1 (HR,95%CI, *P*)	Model 2 (HR, 95%CI *P*)	Model 3 (HR, 95%CI *P*)	Model 4 (HR, 95%CI *P*)
TyG index	2.12 (2.08, 2.17) < 0.001	1.56 (1.52, 1.60) < 0.001	1.60 (1.56, 1.65) < 0.001	1.60 (1.56, 1.65) < 0.001
TyG index (quartile)				
Q1	ref	ref	ref	ref
Q2	1.58 (1.50, 1.66) < 0.001	1.30 (1.24, 1.37) < 0.001	1.34 (1.27, 1.40) < 0.001	1.31 (1.24, 1.37) < 0.001
Q3	2.37 (2.26, 2.48) < 0.001	1.64 (1.56, 1.72) < 0.001	1.71 (1.62, 1.79) < 0.001	1.66 (1.58, 1.74) < 0.001
Q4	3.67 (3.51, 3.83) < 0.001	2.10 (2.00, 2.21) < 0.001	2.23 (2.12, 2.35) < 0.001	2.17 (2.06, 2.29) < 0.001
*P* for trend	< 0.001	< 0.001	< 0.001	< 0.001

Model 1: we did not adjust for any covariates

Model 2: we adjusted for gender, age, SBP, DBP, family history of diabetes, drinking status, smoking status, and BMI

Model 3: we adjusted for gender, age, SBP, DBP, family history of diabetes, drinking status, smoking status, BMI, HDL, LDL-C, AST, ALT, Scr, and BUN

Model 4: All covariates listed in Table 1 were adjusted. However, continuous covariates were adjusted as nonlinearity

*HR* hazard ratios, *CI* confidence interval, *Ref* reference, *TyG index* triglyceride–glucose index

Moreover, the analysis revealed a progressive increase in the HR across the quartiles when using the first quartile (Q1) of TyG as the reference point. Specifically, the HR for the second quartile (Q2) was 1.34 (95%CI 1.27–1.40), for the third quartile (Q3) was 1.71 (95%CI 1.62–1.79), and for the fourth quartile (Q4) was 2.23 (95%CI 2.12–2.35) (Table 4, Model 3).

### The results of sensitivity analysis

A GAM was employed to incorporate the continuity covariate into the analytical equation in the form of a curve. This approach yielded results that were in alignment with those obtained from the fully adjusted model (Table 4, Model 4, HR: 1.60, 95%CI 1.56–1.65). An *E* value of 2.58 indicated a more substantial statistical significance than the relative risk of 1.92 linked to unmeasured confounders and the TyG index. It was inferred that the effect of unknown confounding variables on the TyG index’s association with Pre-DM is minor.

Moreover, a sensitivity analysis was meticulously performed on the subset of participants possessing a BMI below 25 kg/m^2^. This analysis unveiled a sustained positive association between the TyG index and the predisposition towards Pre-DM, persisting even subsequent to the adjustment for potential confounding factors (HR: 1.67, 95%CI 1.61–1.73) (Table 5). Another sensitivity analysis included individuals < 65 years, which also confirmed that the TyG index maintained a positive association with the probability of developing Pre-DM after adjustment for confounders (HR: 1.62, 95%CI 1.58–1.67), as detailed in Table 5. These sensitivity analyses showed the robustness of the findings.

**Table 5:** Relationship between TyG index and prediabetes in different sensitivity analyses

Exposure	Model 5 (HR,95%CI *P*)	Model 6 (HR,95%CI *P*)
TyG index	1.67 (1.61, 1.73) < 0.001	1.62 (1.58, 1.67) < 0.001
TyG index (quartile)		
Q1	ref	ref
Q2	1.34 (1.26, 1.41) < 0.001	1.33 (1.27, 1.41) < 0.001
Q3	1.69 (1.60, 1.79) < 0.001	1.71 (1.62, 1.80) < 0.001
Q4	2.22 (2.09, 2.36) < 0.001	2.27 (2.15, 2.39) < 0.001
*P* for trend	< 0.001	< 0.001

Model 5 was sensitivity analysis in participants with BMI < 25 kg/m^2^. We adjusted gender, age, SBP, DBP, family history of diabetes, drinking status, smoking status, BMI, HDL, LDL-C, AST, ALT, Scr, and BUN

Model 6 was sensitivity analysis in participants aged < 65 years. We adjusted gender, age, SBP, DBP, family history of diabetes, drinking status, smoking status, BMI, HDL, LDL-C, AST, ALT, Scr, and BUN

*HR* hazard ratios, *CI* confidence interval, *Ref* reference, *TyG index* triglyceride–glucose index

### The nonlinear relationship between the TyG index and Pre-DM

**Fig. 3:**
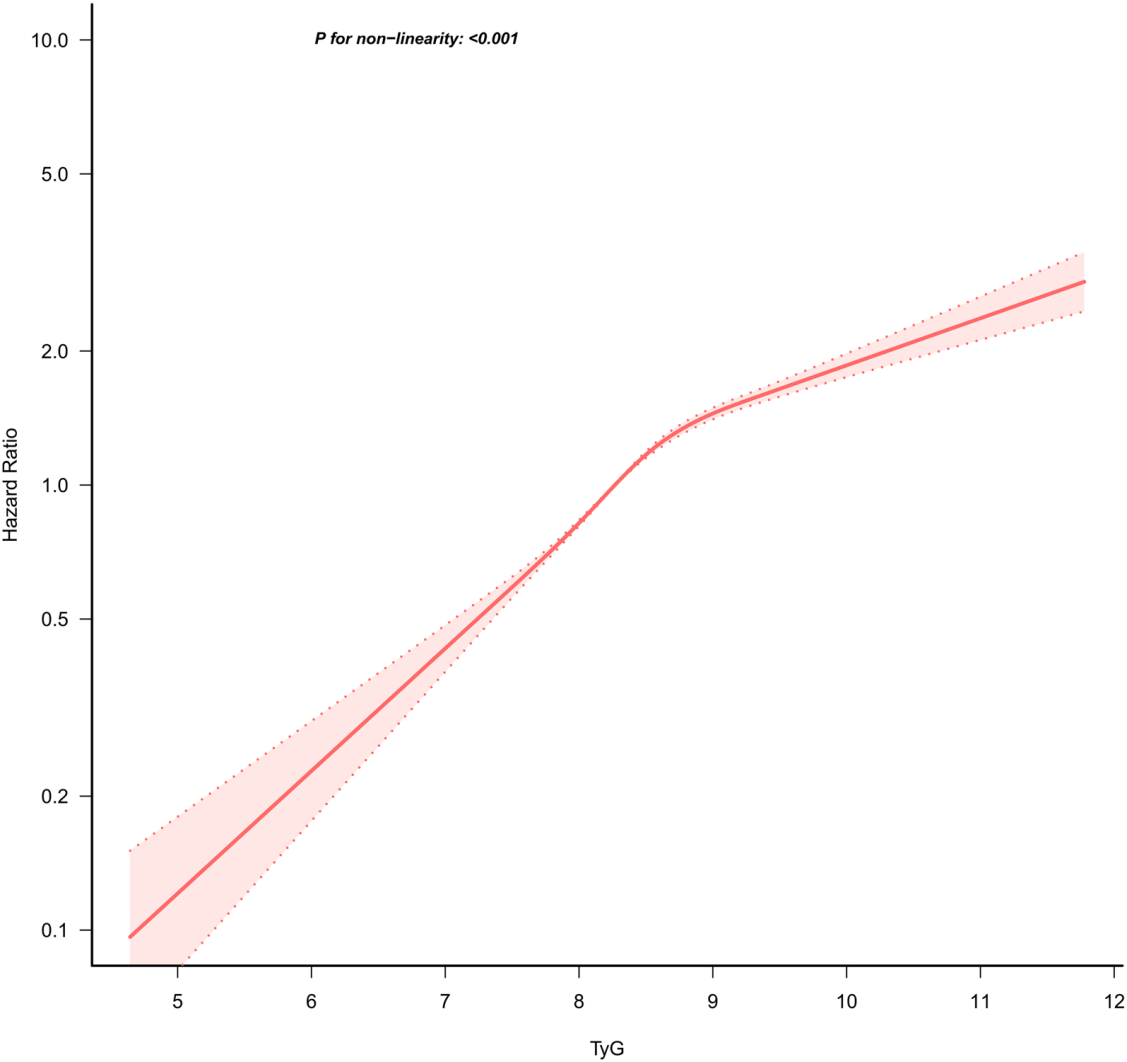
Nonlinear relationship between TyG index and incident prediabetes. A nonlinear relationship between them was detected after adjusting for gender, age, SBP, DBP, family history of diabetes, drinking status, smoking status, BMI, HDL, LDL-C, AST, ALT, Scr, and BUN

Figure 3 reveals a nonlinear correlation between the TyG index and the risk of developing Pre-DM. The *P* value for the nonlinearity test was < 0.001. A two-piecewise Cox proportional hazards regression model pinpointed the TyG index's inflection point at 8.78. The HR was 1.26 (95%CI 1.20–1.33) to the right of the inflection point and 1.94 (95%CI 1.86–2.03) to the left of the inflection point, as shown in Table 6.

**Table 6:** Result of the two-piecewise Cox proportional hazards regression model

Incident prediabetes	HR (95%CI)	P
Fitting model by standard Cox proportional hazards regression	1.60 (1.56, 1.65)	< 0.001
Fitting model by two-piecewise Cox proportional hazards regression		
Inflection points of the TyG index	8.78	
≤ 8.78	1.94 (1.86, 2.03)	< 0.001
> 8.78	1.26 (1.20, 1.33)	< 0.001
*P* for log-likelihood ratio test	< 0.001	

We adjusted for gender, age, SBP, DBP, family history of diabetes, drinking status, smoking status, BMI, HDL, LDL-C, AST, ALT, Scr, and BUN

*HR* hazard ratios, *CI* confidence interval, *Ref* reference, *TyG index* triglyceride–glucose index

### Subgroup analysis

A subgroup analysis was conducted to investigate further potential influences on the TyG index and Pre-DM risk relationship, considering variables such as family history of diabetes, DBP, age, smoking status, BMI, SBP, drinking status, and sex as stratification factors. The analysis identified trends in effect sizes for these factors, as detailed in Table 7. It was found that family history of diabetes, drinking status, and smoking status did not modify the association between the TyG index and Pre-DM risk. The analysis showed a stronger association in subgroups including females, and people with DBP < 90 mmHg, BMI < 25 kg/m^2^, SBP < 140 mmHg, and age < 65 years.

**Table 7:** Effect size of TyG index on prediabetes in prespecified and exploratory subgroups

Characteristic	No of patients	HR (95%CI)	*P* value	P for interaction
Age(years)				
< 65	169,532	1.73 (1.68, 1.78)	< 0.001	< 0.001
≥ 65	9645	1.33 (1.23, 1.44)	< 0.001	
Gender				
Male	95,126	1.49 (1.44, 1.53)	< 0.001	< 0.001
Female	84,051	1.90 (1.82, 1.98)	< 0.001	
Smoking status				
Current-smoker	9311	1.56 (1.42, 1.70)	< 0.001	0.4842
Ex-smoker	2057	1.57 (1.28, 1.92)	< 0.001	
Never-smoker	38,091	1.65 (1.56, 1.75)	< 0.001	
Drinking status				
Current drinker	973	1.70 (1.29, 2.24)	< 0.001	0.2246
Ever drinker	7218	1.49 (1.34, 1.66)	< 0.001	
Never drinker	41,268	1.65 (1.56, 1.75)	< 0.001	
Family history of diabetes				
No	175,640	1.61 (1.56, 1.65)	< 0.001	0.280
Yes	3537	1.49 (1.29, 1.71)	< 0.001	
SBP (mmHg)				
< 140	164,050	1.67 (1.62, 1.72)	< 0.001	< 0.001
≥ 140	15,127	1.37 (1.29, 1.45)	< 0.001	
DBP (mmHg)				
< 90	166,553	1.63 (1.59, 1.68)	< 0.001	0.001
≥ 90	12,624	1.45 (1.36, 1.55)	< 0.001	
BMI (kg/m^2^)				
< 25	132,572	1.82 (1.76, 1.88)	< 0.001	< 0.001
≥ 25	46,605	1.47 (1.42, 1.53)	< 0.001	

1: The above model was adjusted for gender, age, SBP, DBP, family history of diabetes, drinking status, smoking status, BMI, HDL, LDL-C, AST, ALT, Scr, and BUN

2: The model was not adjusted for the stratification variable in each case

## Discussion

This retrospective analysis found a significant correlation between higher TyG index levels and incident Pre-DM. After adjusting for various covariates, the risk of Pre-DM rose by 60% for every unit increase in the TyG index. An inflection point at a TyG value of 8.78 delineated a change in the strength of this association: below this threshold, each unit increase corresponded to a 94% heightened risk of Pre-DM, whereas above it, the risk increase was 26% per unit. Notably, this association was more pronounced in women, individuals younger than 65 years, and those with DBP < 90 mmHg, SBP < 140 mmHg, and BMI < 25 kg/m^2^.

Research has highlighted IR as a primary factor in the development of diabetes and prediabetes, often manifesting well before these conditions are diagnosed [22]. The TyG index has been identified as a preferable method for assessing IR, outperforming other metrics like the homeostasis model assessment of insulin resistance (HOMA-IR) [23]. Investigations in both China and the United States have explored the link between the TyG index and the onset of prediabetes. A notable study involving 4543 individuals, none of whom had prediabetes or diabetes at the start, established a significant positive correlation between the TyG index and incident prediabetes after adjusting for variables, such as lifestyle habits, gender, family medical history, cardiovascular health, age, hypertension, and education (OR: 1.38, 95%CI 1.28–1.48) [14]. Another study, this one with 7,953 subjects, confirmed a strong association between the TyG index and prediabetes risk, factoring in age, sex, systolic blood pressure, medication use, smoking, and alcohol intake (OR: 3.111, 95%CI 2.826–3.425) [15]. Further evidence suggests that the TyG index is more effective than other measures, such as BMI, waist circumference, body roundness index, blood lipid indices, and visceral adiposity index for prediabetes screening [14, 15, 24]. This study supports the growing consensus that high TyG levels are indicative of an increased risk of prediabetes. Unlike prior studies, the results utilized TyG as a categorical and continuous variable to examine its association with prediabetes risk, enhancing precision and reducing information loss in quantifying this relationship. Furthermore, this study distinguished itself by adjusting for a broader range of parameters, including smoking status, levels of ALT, BUN, AST, Scr, FPG, and LDL-C, all of which have been linked to prediabetes development [25–27]. Sensitivity analyses confirmed the persistence of this relationship in participants with a BMI less than 25 kg/m^2^ and those younger than 65 years, underscoring the robustness of the TyG and prediabetes risk connection. Moreover, subgroup analyses revealed notably stronger positive correlations in specific demographics, including women, individuals younger than 65 years or with DBP < 90 mmHg, BMI < 25 kg/m^2^, and SBP < 140 mmHg. The findings from the subgroup analysis underscore the importance of considering the TyG index as an integral part of routine clinical assessments, especially in the identified subgroups of women, younger individuals, and those with normal blood pressure and BMI. Elevated TyG levels in these populations warrant more focused medical attention and proactive measures to prevent the onset of diabetes. By integrating the TyG index into clinical practice, healthcare providers can enhance early detection efforts, personalize intervention strategies, and ultimately reduce diabetes in their patients.

The underlying process through which the TyG index forecasts the risk of prediabetes remains unclear, though IR is acknowledged as a significant factor in this risk. It is hypothesized that IR mediates the association between the TyG index and prediabetes risk due to TyG’s pronounced capability to predict IR. For instance, within a Mexican cohort, the TyG index demonstrated a sensitivity of 96.5% for IR prediction, closely rivaling the hyperinsulinemic–euglycemic clamp, the benchmark for IR assessment [12]. This high predictive value of TyG for prediabetes can be attributed to IR being a fundamental cause of glucose regulation abnormalities. The TyG index is a combined measure of triglyceride levels and fasting blood glucose. Triglycerides break down into glycerol and fatty acids, with an increase in free fatty acids being transported from adipose to non-adipose tissues, thereby fostering IR. Elevated triglycerides facilitate the movement of high levels of free fatty acids to the liver, augmenting glucose production and specifically enhancing gluconeogenesis [28]. Elevated triglyceride and glucose levels exacerbate chronic low-grade inflammation, characterized by increased pro-inflammatory cytokines such as TNF-α and IL-6 [29–31]. This inflammatory state facilitates ectopic fat deposition in non-adipose tissues, impairing insulin signaling and inducing oxidative stress [32, 33]. Furthermore, oxidative stress, resulting from an imbalance between the production of reactive oxygen species (ROS) and the body’s antioxidant defenses, contributes to β-cell dysfunction. Elevated free fatty acids, combined with high lipid levels, increase the generation of ROS, causing oxidative damage to pancreatic β-cells [34]. This oxidative stress not only impairs β-cell functionality but also diminishes insulin secretion in response to glucose [35, 36].

This study revealed a nonlinear association between the TyG index and Pre-DM, even after adjusting for factors such as family history of diabetes, DBP, age, smoking status, BMI, SBP, drinking status, sex, HDL-C, BUN, ALT, Scr, LDL-C, and AST. This study identified an inflection point in the TyG index using a two-piecewise Cox proportional hazards regression model. Below the TyG index of 8.78, each unit increase in the TyG index correlated with a 94% heightened risk of developing Pre-DM. Above the TyG index of 8.78, the risk increase per unit rise in the TyG index moderated to 26%. This identification of a curvilinear relationship underscores the TyG index’s critical role in clinical assessments, offering a valuable tool for enhancing consultations and refining strategies for prediabetes prevention.

This study presents several key strengths. First, it delved into the nonlinear connection between the TyG index and Pre-DM. Second, the study employed rigorous statistical methods to minimize the impact of residual confounding factors. Third, comprehensive sensitivity analyses were undertaken to substantiate the consistency and reliability of the findings. These analyses encompassed the conversion of the TyG index into categorical variables, the application of GAM for modeling the continuity covariate as a non-linear curve, and the computation of *E* values to evaluate the potential influence of unmeasured confounders. Furthermore, this study reassessed the TyG-Pre-DM relationship after removing participants with a BMI of 25 kg/m^2^ or higher or those aged 65 years and above. Finally, subgroup analyses were performed to evaluate specific confounding factors that could affect the TyG index and Pre-DM linkage.

This study is subject to several limitations. First, the potential underestimation of Pre-DM due to the absence of glycated hemoglobin measurements and the 2-h oral glucose tolerance test must be acknowledged. In future research, we aim to incorporate a more comprehensive set of variables, including HbA1c and the 2-h OGTT, to elucidate the relationship between the TyG index and prediabetes more robustly. Second, despite diligent adjustments for recognized confounders, this study did not account for or measure several variables influencing prediabetes, such as waist-to-height ratio, sleep duration, dietary patterns, lipid-modifying treatments, and physical activity. Previous research has demonstrated the significance of these factors in the development of prediabetes [37–39]. The absence of these factors may influence the comprehensiveness and accuracy of the findings. However, the application of *E* values in the analysis suggests that the likelihood of these unmeasured confounders significantly altering the findings is low. Future studies could benefit from a more inclusive approach that integrates a broader array of variables, including waist-to-height ratio, sleep duration, dietary patterns, lipid-modifying treatments, and exercise specifics, through enhanced study protocols or collaboration with other research initiatives. Third, this study lacked weighted data, so this study could not calculate weighted results. Fourth, there were instances of missing data in this study, particularly concerning HDL-C and LDL-C values. Although multiple imputation techniques were employed to address the missing data, it is acknowledged that such gaps may result in unstable results. In future studies, we will endeavor to collect complete data sets to mitigate the limitations posed by missing values and enhance the reliability of the findings. Fifth, the TyG index was measured only at the study’s outset, without accounting for potential variations over time. In future iterations of this study, incorporating longitudinal data on TyG index fluctuations and employing models such as the GAM could provide deeper insights into how changes in the TyG index influence the risk of developing Pre-DM.

## Conclusion

This research demonstrated a positive, non-linear correlation between the TyG index and the likelihood of developing Pre-DM among Chinese adults. Specifically, a TyG index below 8.78 was significantly linked to an increased risk of Pre-DM. The findings of this study provide valuable insights for enhancing clinical consultations and refining strategies for the prevention of Pre-DM in patients.

## Supplementary Information

Additional file 1.

## Data Availability

No datasets were generated or analysed during the current study.
