# Supplementary material for: Non-linear connection between the triglyceride–glucose index and prediabetes risk among Chinese adults: a secondary retrospective cohort study
Source: Eur J Med Res. 2024 Nov 5;29:529. doi: 10.1186/s40001-024-02121-x (PMC11536673; doi:10.1186/s40001-024-02121-x)
Supplement: Supplementary file 1 — Additional file 1. [file 40001_2024_2121_MOESM1_ESM.docx]

**Table S1** The results of the collinearity screening

|  | Step 1 | Step 2 |
| --- | --- | --- |
| Gender | 2.6 | 2.6 |
| Age(years) | 1.3 | 1.3 |
| Drinking status | 1.1 | 1.1 |
| Smoking status | 1.3 | 1.3 |
| Family history of diabetes | 1 | 1 |
| SBP (mmHg) | 2.1 | 2.1 |
| DBP (mmHg) | 2 | 2 |
| BMI (kg/m^2^) | 1.5 | 1.4 |
| ALT (U/L) | 3.2 | 3.2 |
| AST (U/L) | 2.9 | 2.9 |
| HDL-C (mmol/L) | 1.4 | 1.2 |
| LDL-C (mmol/L) | 5.4 | 1.1 |
| TC (mmol/L) | 5.9 | NA |
| BUN (mmol/L) | 1.2 | 1.2 |
| Scr (umol/L) | 2.1 | 2.1 |

**Table S2** Relationship between the TyG index and prediabetes in different models

| Variable | Model 1 (HR.,95% CI, P) | Model 2 (HR,95% CI, P) | Model 3 (HR,95% CI, P) |
| --- | --- | --- | --- |
| TyG index | 2.12 (2.08, 2.17) <0.0001 | 1.56 (1.52, 1.60) <0.0001 | 1.60 (1.56, 1.65) <0.0001 |
| TyG index (quartile) |  |  |  |
| Q1 | ref | ref | ref |
| Q2 | 1.58 (1.50, 1.66) <0.0001 | 1.30 (1.24, 1.37) <0.0001 | 1.33 (1.26, 1.40) <0.0001 |
| Q3 | 2.37 (2.26, 2.48) <0.0001 | 1.64 (1.56, 1.72) <0.0001 | 1.70 (1.62, 1.79) <0.0001 |
| Q4 | 3.67 (3.51, 3.83) <0.0001 | 2.10 (2.00, 2.21) <0.0001 | 2.22 (2.11, 2.34) <0.0001 |
| P for trend | <0.0001 | <0.0001 | <0.0001 |

Model 1: we did not adjust for any covariates

Model 2: we adjusted for gender, age, SBP, DBP, family history of diabetes, and BMI.

Model 3: we adjusted for gender, age, SBP, DBP, family history of diabetes, BMI, HDL, LDL-C, ALT, Scr, and BUN.

**Table S3** Relationship between the TyG index and prediabetes in different models

| Variable | Model 1 (HR.,95% CI, P) | Model 2 (HR,95% CI, P) | Model 3 (HR,95% CI, P) |
| --- | --- | --- | --- |
| TyG index | 2.11 (1.98, 2.26) <0.0001 | 1.59 (1.47, 1.72) <0.0001 | 1.61 (1.49, 1.75) <0.0001 |
| TyG index (quartile) |  |  |  |
| Q1 | ref | ref | ref |
| Q2 | 1.54 (1.31, 1.82) <0.0001 | 1.27 (1.07, 1.49) 0.0051 | 1.29 (1.09, 1.52) 0.0030 |
| Q3 | 2.28 (1.96, 2.65) <0.0001 | 1.59 (1.35, 1.86) <0.0001 | 1.63 (1.39, 1.91) <0.0001 |
| Q4 | 3.74 (3.25, 4.31) <0.0001 | 2.18 (1.86, 2.55) <0.0001 | 2.26 (1.91, 2.66) <0.0001 |
| P for trend | <0.0001 | <0.0001 | <0.0001 |

Model 1: we did not adjust for any covariates

Model 2: we adjusted for gender, age, SBP, DBP, family history of diabetes, drinking status, smoking status, and BMI.

Model 3: we adjusted for gender, age, SBP, DBP, family history of diabetes, drinking status, smoking status, BMI, HDL, LDL-C, AST, ALT, Scr, and BUN.

Note: We excluded populations with missing data on smoking status, drinking status, and AST.

**Table S4** Relationship between the TyG index and prediabetes in different models

| Variable | Model 1 (HR.,95% CI, P) | Model 2 (HR,95% CI, P) | Model 3 (HR,95% CI, P) |
| --- | --- | --- | --- |
| TyG index | 2.12 (2.08, 2.17) <0.0001 | 1.60 (1.52, 1.68) <0.0001 | 1.59 (1.43, 1.77) <0.0001 |
| TyG index (quartile) |  |  |  |
| Q1 | ref | ref | ref |
| Q2 | 1.58 (1.50, 1.66) <0.0001 | 1.25 (1.13, 1.38) <0.0001 | 1.35 (1.08, 1.69) 0.0088 |
| Q3 | 2.37 (2.26, 2.48) <0.0001 | 1.56 (1.42, 1.72) <0.0001 | 1.61 (1.29, 2.00) <0.0001 |
| Q4 | 3.67 (3.51, 3.83) <0.0001 | 2.12 (1.92, 2.33) <0.0001 | 2.28 (1.82, 2.84) <0.0001 |
| P for trend | <0.0001 | <0.0001 | <0.0001 |

Model 1: we did not adjust for any covariates

Model 2: we adjusted for gender, age, SBP, DBP, family history of diabetes, drinking status, smoking status, and BMI.

Model 3: we adjusted for gender, age, SBP, DBP, family history of diabetes, drinking status, smoking status, BMI, HDL, LDL-C, AST, ALT, Scr, and BUN.

Note: We excluded populations with missing data.
